# Intrathoracic synovial sarcoma with BRAF V600E mutation

**DOI:** 10.18632/oncotarget.28475

**Published:** 2023-07-07

**Authors:** Ida Russo, Sabina Barresi, Pier Luigi Di Paolo, Valentina Di Ruscio, Giada Del Baldo, Annalisa Serra, Silvia Vallese, Evelina Miele, Angela Mastronuzzi, Rita Alaggio, Andrea Ferrari, Giuseppe Maria Milano

**Affiliations:** ^1^Department of Pediatric Hematology and Oncology, Gene and Cellular Therapy, Bambino Gesù Children's Hospital IRCCS, Rome, Italy; ^2^Department of Pathology, Bambino Gesù Children's Hospital IRCCS, Rome, Italy; ^3^Pediatric Oncology Unit, Fondazione IRCCS Istituto Nazionale Tumori, Milano, Italy; ^4^Department of Radiology, Bambino Gesù Children's Hospital IRCCS, Rome, Italy

**Keywords:** synovial sarcoma, next-generation sequencing, BRAF V600E mutation, targeted therapy

## Abstract

We report a case of 15-year-old boy with intrathoracic synovial sarcoma who relapsed after standard chemotherapy, surgery and radiotherapy. The molecular analysis of the tumour identified a BRAF V600E mutation at time of progression of relapsed disease under third line systemic treatment. This mutation is commonly seen in melanomas and papillary thyroid cancers, but less prevalent (typically <5%) across a variety of other cancer types. The patient underwent selective BRAF inhibitor Vemurafenib treatment achieving partial response (PR) with a progression free survival (PFS) ratio of 1.6 months and an overall survival of 19 months, alive in continuous PR.

This case highlights the role of routinely next generation sequencing (NGS) used to drive treatment choice and to investigate extensively synovial sarcoma tumour for BRAF mutation.

## INTRODUCTION

Synovial sarcoma (SS) is a highly malignant mesenchymal tumour that occurs mainly in adolescents and young adults [1], genetically defined by SS18 gene fusions including SS18–SSX1, SS18–SSX2, and SS18–SSX4. The SS18–SSX protein exerts oncogenic activity through various mechanisms that disrupt epigenetic control. The fusion protein binds to the SWI/SNF chromatin remodelling complex, resulting in the displacement of native SS18 and eviction of BAF47 (SMARCB1) [2, 3]. The fusion protein also interacts with KDM2B to bring together the SWI/ SNF complex and PRC1.1 on the unmethylated CpG islands to aberrantly reactivate the expression of developmentally regulated genes that are otherwise repressed by PRC2 [4, 5]. SS is otherwise genomically silent and additional genetic alterations are rare in the primary tumours, with uncommon secondary mutations including TP53, PTEN, CTNNB1, APC, SETD2, and FBXW7 [6].

The treatment of SS is multimodal, involving surgery, radiotherapy and chemotherapy. While the overall prognosis is generally quite satisfactory in children and adolescents with localised synovial sarcoma at diagnosis [7], the outcome remains poor for patients who relapse, with a reported 5-year post relapse survival around 30% [8]. Conversely to the front-line standardized treatment options [9], patients with relapse generally have an individualized approach, and to date there is a lack of consensus about standard treatment approaches for the subset of relapsing patients with SS, both in adults and in children. As a consequence, there is a strong need for novel strategy and new effective agents.

Here we present the case of 15-year-old boy with intrathoracic SS who relapsed after standard chemotherapy, surgery and radiotherapy: molecular analysis identified a BRAF V600E mutation at progression of relapsed disease under third line systemic treatment. The patient received targeted treatment with a selective BRAF inhibitor as individual treatment.

## CASE REPORT

**Figure 1:**
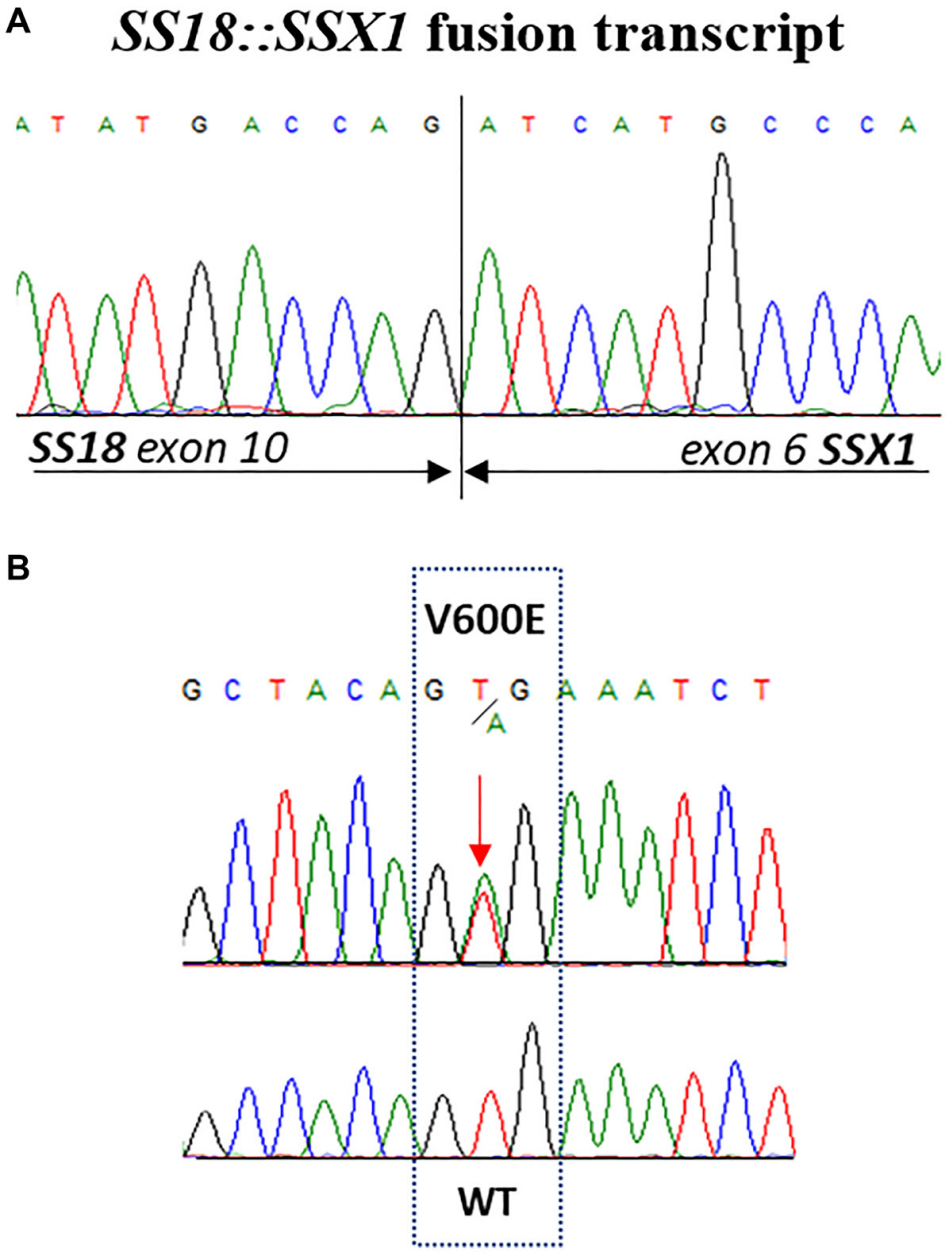
(**A**) Sequence analysis of SYT-SSX transcripts obtained from synovial sarcoma biopsy. (**B**) Partial electropherogram showing the activating missense mutation in codon 600 of exon 15 (V600E) of BRAF gene identified in tumour resection of patient’s synovial sarcoma.

**Figure 2:**
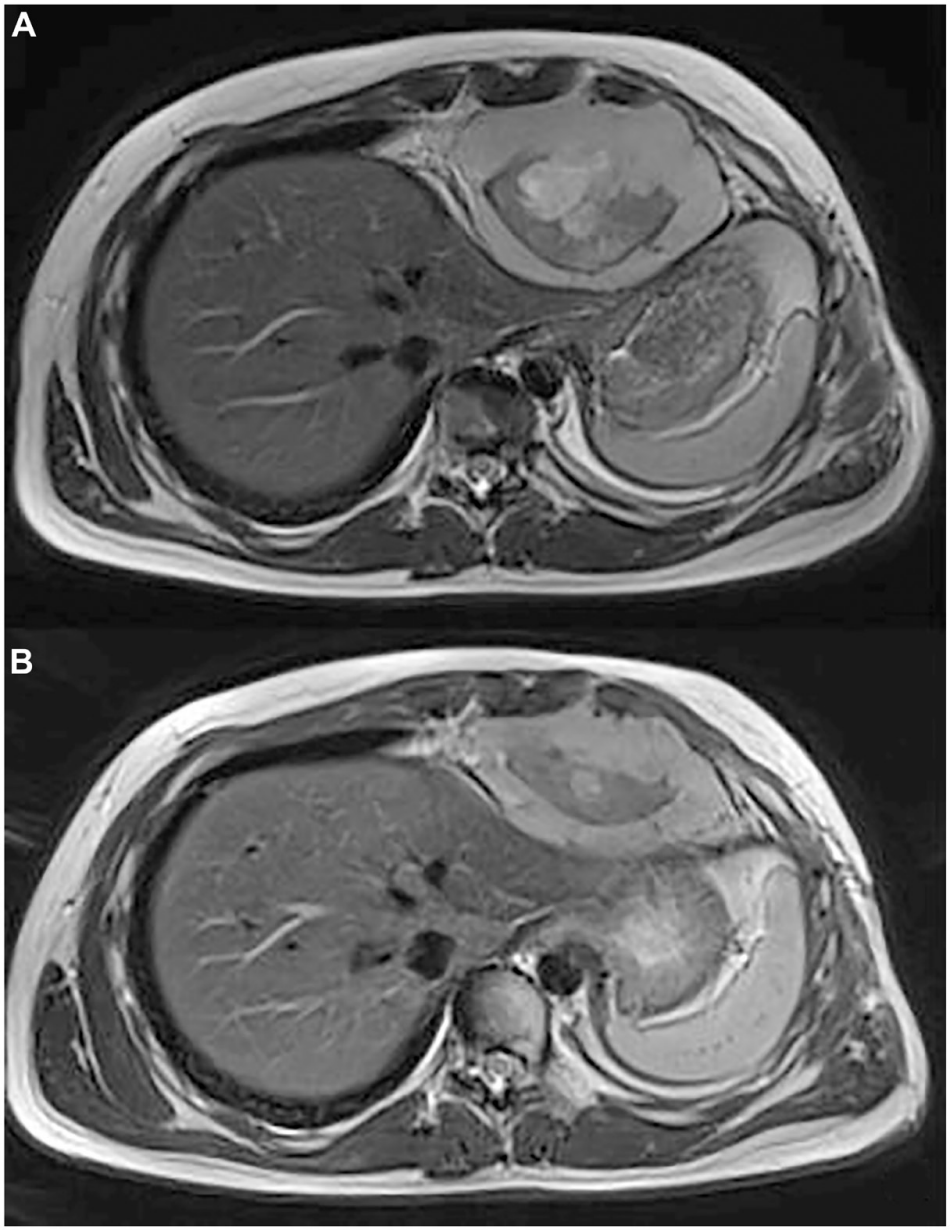
(**A**) Axial T2 MR shows a heterogeneous mass in the cardiophrenic space with evidence of anterior chest wall invasion and intercostal invasion. (**B**) Axial T2 MR shows 35 % of reduction in size of the mass in the cardiophrenic space.

A 15-year-old boy was diagnosed has having intrathoracic SS, diagnosis was confirmed by *SS18::SSX1* fusion transcript detected by reverse-transcriptase polymerase chain reaction (Figure 1A). Staging work-up of the disease did not show any distant metastases. The patient received chemotherapy with 3 cycles of doxorubicin and ifosfamide, 1 cycle of ifosfamide, which resulted in partial response. The patient underwent tumour resection with left upper lung lobe, parietal and pericardial pleura. Subsequently he received adjuvant chemotherapy, with 1 cycle of ifosfamide, and consolidation local radiotherapy (RT) with Volumetric Modulated Arc Therapy technique (VMAT), total dose of 50.4 Gy in daily fractions of 1.8 Gy. One month after RT, a local relapse was found on magnetic resonance image (MRI) without distant lesions. Based on the strength of reported efficacy in phase III trial [10], patient was therefore treated with multitargeted tyrosine kinase inhibitor Pazopanib monotherapy at dose of 800 mg QD, discontinued 72 hours later for acute myopericarditis possible related to RT late effects. After resolution of the cardiotoxicity, a third-line nucleoside metabolic inhibitor Gemcitabine (750 mg/mq/dose) every two weeks was started, as an alternative treatment which may be considered in patients affected by SS who cannot tolerate or are resistant to standard chemotherapy [11]. We chose to omit docetaxel considering previous toxicities. After 4 courses of Gemcitabine, a new MRI showed intrathoracic tumour progression and a new lesion appearance at the homolateral thoracic wall. Patient was referred to our internal molecular tumour board (MTB). In order to identify molecular target for cancer, the TruSight Oncology 500 panel targeting 523 cancer-relevant genes on tumour resection has been performed. Next generation sequencing (NGS) analysis showed low tumour mutational burden (TMB) and stable microsatellite instability (MSI) status and detected a *BRAF* p.V600E (c.1799T>A) mutation with a variant allele frequency (VAF) of 49% in a sample with histologically estimated tumour cell ratio of 85% (Figure 1B). The mutation *BRAF* p.V600E was also investigated and confirmed in PCR on the post-therapy tumour biopsy. Vemurafenib, at initial dose of 240 mg BID was started in low dose regimen taking into consideration the toxicity profile of the drug and the previous cardiological and pulmonary patient’s morbidities. Despite starting low dosage of the drug, radiological examination after 4 months unexpectedly showed partial response (PR) for both intrathoracic and chest wall lesions, according to RECIST version 1.1 [12], with volume shrinkage of the intrathoracic lesion of 35% (Figure 2A, 2B). The dosage at 480 mg BID was increased and well tolerated. Patient experienced only G1 folliculitis, G2 cough, G1 asthenia, G1 hyperuricemia. Toxicity was graded according to the National Cancer Institute’s Common Toxicity Criteria, version 5.0 [13]. Patient is currently alive with a progression free survival (PFS) of 6 months and an overall survival (OS) of 19 months.

## DISCUSSION

The prognosis of recurrent/metastatic SS remains poor, highlighting the need for a novel therapeutic strategy. According to literature data, major strategy at relapse includes radical surgical approach, where feasible; still debated is the role of radiotherapy and second-line chemotherapy [8, 14, 15]. After the first relapse early treatment with tyrosine kinase inhibitor pazopanib was started, on the strength of reported efficacy in phase III trial. Notably in the phase III registration study with Pazopanib, SS and leiomyosarcoma were the two histologies with the best PFS (the primary endpoint of the study) [10]. Unfortunately, our patients poorly tolerated antiangiogenic treatment, by developing myopericarditis, likely due to post-actinic cardiological toxicity. The drug was therefore discontinued and followed by nucleoside metabolic inhibitor gemcitabine as third-line therapy in soft tissue sarcoma (STS) with poor prognosis [11]. Given the evidence of local disease progression under Gemcitabine, according with our practice, NGS test was done and unexpectedly BRAF V600E mutation was found, therefore a BRAF inhibitor was started.

BRAF V600E mutations, common in melanomas and papillary thyroid cancers, in approximately 50% of tumour specimens, occur with much lower frequency (typically <5%) across a variety of other cancer types, including colorectal carcinoma, glioma, non-small cell lung carcinoma, cholangiocarcinoma and several hematologic malignancies [16]. The selective BRAF inhibitor Vemurafenib is an oral small molecule approved by FDA in 2011 and the first successful therapy targeting BRAF-mutated melanoma [17]. In literature three histology-independent “basket” researches were reported, exploring the efficacy of Vemurafenib in BRAF V600E mutant non-melanoma cancers [18, 19], in which 2 SS (among 6 STS patients) with BRAF V600E mutation enrolled, and one report of 2 patients with SS expressing BRAF V600E mutation [20]. Our patient is the fifth one in the current literature affected by BRAF V600E mutated SS. The disease characteristics and treatment details of the other four patients are summarized in Table 1.

**Table 1:** SS patients with BRAF V600E mutation

Author (year) [References]	Sex, age	Tumour characteristics at onset	First-line treatment	Relapses/further line treatments	Targeted th. with BRAF +/− MEK inhibitors	Response to Targeted Th.	PFS/Outcome
Sho Watanabe (2020) [17]	f, 32y	Right thoracic cavity mass (12 cm diameter), N0, M0. Histology: monophasic SS with a classic spindle cell morphology. Fusion transcript SS18–SSX2	Neoadjuvant Ifosfamide-Doxorubicin CT regimen; Surgery; adjuvant Ifosfamide-Doxorubicin CT regimen	Pulmonary metastasis after 18 months/ - Trabectedin in phase 1 trial; - Ifosfamide monotherapy; - Pazopanib	Not done	N.A.	N.A./Died of the disease 43 months after diagnosis.
Sho Watanabe (2020) [17]	f, 23y	Superior mediastinum mass (4.3 cm diameter), concomitant hemothorax, N0, M0. Histology: monophasic SS with a classic spindle cell morphology. Fusion transcript SS18–SSX2	Surgery	Right arm and shoulder local recurrence after 5 months/ - Ifosfamide-Doxorubicin CT regimen; - Local radiation therapy; - Pazopanib; - Ifosfamide monotherapy. -Transient tumour response with the subsequent regrowth	Dabrafenib 150 mg BID + Trametinib 2 mg QD	CR	7.5 months/Local recurrence (acquired NRAS-Q61K mutation in tumour specimen)/N.A.
Vivek Subbiah (2020) VE-Basket trial [16]	f, 59y	N.A.	Vemurafenib	N.A.	Vemurafenib 960 mg BID	PD	1.6 months/Died of the disease 2 months after diagnosis.
Vivek Subbiah (2020) VE-Basket trial [16]	m, 47y	N.A.	N.A.	N.A.	Vemurafenib 960 mg BID	PD	1.2 months/Died of the disease 3.7 months after diagnosis.
Our case	M 15y	Left thoracic cavity mass, Nx, M0. Histology: monophasic SS with a classic spindle cell morphology. Fusion transcript SS18–SSX1	Neoadjuvant Ifosfamide-Doxorubicin CT regimen; Surgery; adjuvant Proton therapy Ifosfamide- CT regimen	- Pazopanib; Gemcitabine/Docetaxel	Vemurafenib 240 mg BID after 4 months Increased to 480 mg BID	PR	6 months/ Alive at 19 months after diagnosis.

Abbreviations: SS: Synovial Sarcoma; PFS: progression-free survival; f: female; m: male; y: years; CT: chemotherapy; th: therapy; N.A.: not available; N: node; M: metastasis; BID: twice a day; QD: once a day; CR: complete response; PD: progressive disease.

Among patients reported in the literature, affected by SS harboring BRAF V600E mutation, all patients but one were female, aged between 23–59 years old. Two patients presented unresectable thoracic large tumours, monophasic spindle cell histology, SS18-SSX2 fusion positive (for the other 2 patients, disease characteristics were not available). All patients relapsed after prior line treatments, and all but one received targeted therapy with BRAF inhibitors (in one case, associated with MEK inhibitor Trametinib). The best response was a complete response lasting 7.5 months. Similarly, our patient has intrathoracic SS, unresectable at diagnosis and relapsing/refractory after several lines of treatment. Vemurafenib is a drug with tolerable safety profile. In early studies, the most frequently encountered grade 2 or 3 side effects included cutaneous squamous cell carcinoma, fatigue, arthralgia, rash, nausea, photosensitivity, pruritus, and palmar-plantar dysesthesia. The clinical data on Vemurafenib in chemotherapy-refractory SS is very limited. Except for the above-mentioned 4 patients reported, the writer has never come across any similar cases. PFS ratio (PFS1 during or after molecular targeted therapy/PFS2 during or after the last prior systemic therapy) is utilized to assess the efficacy of molecularly guided treatment, and in recent studies, some fixed PFS ratios such as 1.3 are often regarded as thresholds for the evaluation of clinical benefit [21, 22]. The PFS ratio of our reported patient is 1.6, highlighting the benefit of NGS in selecting personalized regimens for SS patients whose disease has progressed after multiple therapies. Our data highlight the importance of implementing molecular tests in SS patients to evaluate BRAF mutational actual incidence in these neoplasms.

### Institutional review board statement

The case report was approved by the Institutional Review Board of Bambino Gesù Children’s Hospital.

## Abbreviations

SS: Synovial Sarcoma
RT: Radiotherapy
VMAT: Volumetric Modulated Arc Therapy Technique
MRI: Magnetic Resonance Image
MTB: Molecular Tumour Board
MSI: Microsatellite Instability
NGS: Next Generation Sequencing
TMB: Tumour Mutational Burden
VAF: Variant Allele Frequency
PR: Partial Response
PFS: Progression Free Survival
OS: Overall Survival
